# Allergen extract outperforms molecular components in basophil activation test in a pediatric cohort

**DOI:** 10.3389/fimmu.2025.1701351

**Published:** 2026-01-20

**Authors:** Alexandre Chhing, Simone Choi, Dounia Khelifi-Touhami, Aïcha Abbas, Eric Ballot, Nathalie Cottel, Sarah Saf, Garance Germain, Nathalie Lambert, Pierre Challier, Sylvie Pauliat, Anaïs Lemoine, Melisande Bourgoin-Heck, Jocelyne Just, Stéphanie Wanin, Yannick Chantran

**Affiliations:** 1 Immunology Department, St-Antoine & Trousseau Hospital, Sorbonne University, AP-HP, Paris, France; 2 Health Environmental Risk Assessment (HERA) Team, UMR261 MERIT, IRD, Inserm 1344, Faculté de Pharmacie, Université Paris Cité, Paris, France; 3 Allergology Department, Trousseau Hospital, Sorbonne University, AP-HP, Paris, France; 4 Paediatric Nutrition and Gastroenterology Department, Trousseau Hospital, Sorbonne University, AP-HP, Paris, France

**Keywords:** allergy diagnosis, basophil activation test, biomarkers, method comparison, molecular allergens

## Abstract

**Background:**

The use of molecular allergens have greatly improved the clinical relevance of specific IgE serologies during allergy work-up. Very little is known regarding the added value of molecular allergens in the results of the basophil activation test (BAT).

**Objective:**

To study the BAT concordance when using similar amounts of molecular allergens against source extracts, and to assess factors associated with BAT discrepancies between extract and related component.

**Methods:**

Systematic retrospective monocentric study of all BAT performed at the Trousseau Hospital, Paris, France, with both molecular components and corresponding extract.

**Results:**

Data from 213 interpretable extract/component BAT pairs (89 cow milk, 70 wheat, 28 house dust mites (HDM), 12 peanut, 9 hen egg, 3 peach, 1 apple, 1 chicken meat), corresponding to 150 blood samples from 109 patients were analyzed. Among BAT pairs showing allergen sensitizations, the two reagents only showed moderate agreement (κ = 0.62), with 18% (36/201) discordant BAT results. Among 36 cases of discrepant BAT results between extract and molecular component, 69% (25/36) were extract^+^/component^–^ (p=0.03), in line with genuine allergic status, in contrast with the opposite case. Even in extract^+^/component^+^ concordant cases, component-stimulated basophils showed 6% less activation rates than their extract-stimulated counterpart. After stratification by allergen, better performances were confirmed with cow milk extract, but not with other allergens, which display large disparity regarding the propensity of different molecular component to activate basophils. Independently of the considered allergen, other variables such as total IgE, BAT positive control values, and treatment with monoclonal antibody or allergen-specific immunotherapy, appear to further modulate the risk of presenting a discordant BAT result.

**Conclusion:**

In our study population, molecular components had lower capacity than the corresponding extract to activate IgE-sensitized basophils, which partly explain higher rates of true positive extract^+^/component^–^ BAT. Component-based BAT can potentially lead to false-negative results, and extract-based should generally be preferred, especially for cow milk.

## 1 Introduction

In the past decade, the basophil activation test (BAT) has emerged as a powerful surrogate marker of provocation tests for allergy diagnosis (1, 2). This led to the recent implementation of BAT into international recommendations for the diagnostic work-up of different food allergies (3). In addition, further studies suggest that BAT might contribute to stratify patients with high risk of severe reactions, or low eliciting dose (4, 5), and to monitor tolerance acquisition (6).

In the meantime, specific IgE (sIgE) against molecular components have also been a game-changer in the exploration of allergy, providing comprehensive insights into primary Vs. cross-sensitization, risk of allergic reaction among sensitized individuals, prediction of severe or persistent allergic reactions (7).

Thus, it is tempting to assume that BAT would greatly benefit from the use of molecular components in place of allergenic source extracts (8). However, as an *ex vivo* functional test, BAT is expected to mimic real-life conditions, where an isolated molecular allergen is never to be encountered. In addition, BAT results might integrate additional factors over mere IgE sensitizations, such as patient’s basophils reactivity, balance between relevant and other membrane-bound IgEs, and the presence of circulating neutralizing immunoglobulins such as allergen-specific IgG4. Beyond these speculative considerations, there is very little evidence to date which directly compare the performances of extract-based and component-based BAT.

The present study is a retrospective monocentric systematic study aiming to compare the respective performances of allergen extracts and molecular components of similar sources regarding basophil activation and diagnostic performances of the BAT.

## 2 Materials & methods

### 2.1 Study design and participants

This retrospective monocentric study systematically reviewed all BAT performed in a pediatric university-based outpatient practice, from January 2017 to May 2025, at the Trousseau Hospital in Paris, France. Inclusion criteria were: having a BAT prescribed and performed with both a source protein extract and at least one molecular allergen from this source. Exclusion criteria were: negative control > 15% CD63^+^ basophils; positive control corresponding to the high affinity IgE Fc-fragment Receptor (FcεRI)-activated < 15% CD63^+^ basophils (5). All the procedures reflected routine patient care at the study center. The protocol was endorsed by the direct procedure of the Institutional Review Board of the Medical Ethics Committee on Research of AP-HP (https://recherche.aphp.fr/eds).

### 2.2 Basophil activation test

The BAT was carried oud as routine care test, using the FlowCAST assay kit (Bühlmann Laboratories, Schönenbuch, Switzerland) and according to the manufacturer’s recommendations from the FK-CCR supplier procedure. In brief, after gentle stirring, 50 µL of whole blood (EDTA) was incubated with 50 µL of allergen solution at 100, 10, and 1 ng/mL (22.5, 2.25, and 0.225 ng/mL in stimulation buffer, respectively) for 15 minutes at 37 °C. Ara h 2 was tested at 20, 2, and 0.2 ng/mL (4.5, 0.45, and 0.045 ng/mL in stimulation buffer), according to the manufacturer’s recommendations. Control conditions included 50 µL of activation buffer (negative control) and 50 µL of anti-FcϵRI antibody solution or fMLP solution (positive controls with nonspecific stimulation). Basophils were gated based on CCR3^+^/SSC^low^ window on the negative control, and activated basophils on CCR3^+^/CD63^+^ window on the FcϵRI-positive control. At least 500 basophils were analyzed for each condition. The percentage of activated basophils was measured for each experimental condition. All allergens were purchased from BAT kit manufacturer’s, except from Gal d 1, Gal d 2, Der p 1, Der f 1, Pru p 3 purchased from Indoor Biotechnologies Inc. (Charlottesville, Virginia, USA), and Gal d 7, Pru p 7 from Creative Biomart (Shirley, NY, USA). Peach, apple, and chicken meat extracts were homemade in our laboratory.

### 2.3 Demographic and clinical data

Demographic and clinical data were collected from lab and clinical charts. Collected demographic characteristics were: age, and sex. Collected clinical characteristics were: allergic status based on unequivocal anamnesis or open provocation test results; presence, type and duration of monoclonal antibody (mAb)-based immunotherapy, and presence and duration of allergen-based immunotherapy.

### 2.4 Serological parameters

Serological parameters were collected from serum samples paired with BAT whole blood collection, which is standard care practice in our laboratory. Collected serological parameters were: total IgE, extract- and component- specific IgE (sIgE), extract- and component specific IgG4. Total IgE, sIgE and specific IgG4 were evaluated by ImmunoCAP on a Phadia250 instrument (ThermoFisher Scientific, Uppsala, Sweden), in accordance with manufacturer’s recommendations and routine practices of our certified laboratory.

### 2.5 Statistical analysis

The concordance between extract-based and component-based BAT was evaluated by Cohen’s kappa coefficient. The interpretation of the kappa coefficient follows McHugh’s interpretation (9): agreement was considered nul, minimal, weak, moderate, strong, and almost perfect, if Cohen’s kappa was 0 – 0.2, 0.2 – 0.4, 0.4 – 0.6, 0.6 – 0.8, 0.8 – 0.9, > 0.9, respectively. The McNemar’s test was used to analyze unbalanced disagreement between methods, also conveyed as odds ratios (OR) and associated 95% confidence intervals (95% CI). Correlations between extract- and component-stimulated basophil activation rates with similar concentrations of allergens at all tested dilutions were performed among concordant positive BAT using non-parametric Kendall’s Tau-b correlation. Bias toward systematic difference regarding basophil activation by similar amounts of extract and components was assessed by paired Wilcoxon’s test. A multivariable linear regression model was used to stratify the difference between extract- and component-stimulated basophil activation rates according to the investigated allergenic source. Analysis of other demographical, clinical, and biological factors associated with extract/component BAT dissociation was carried out using multivariable logistic regression models. All the tests were two-sided, with significant p-values below type I error risk α=0.05. Analyses were conducted using R software v4.3.3 (R Foundation for Statistical Computing, Vienna, Austria).

## 3 Results

### 3.1 Data selection

Results of BAT performed between Jan. 2017 to May 2025 in the Immunology Dpt. associated with the Trousseau pediatric hospital, Paris, France, were systematically reviewed. Overall, 2588 samples were addressed from tertiary allergy units to our laboratory, corresponding to 1545 unique subjects, and to 4511 tested allergens. From these database, the study included BAT results from 213 extract/component pairs, corresponding to 150 samples from 109 patients. Eight samples were tested with more than one allergen source.

The most frequent extract/component pair was cow milk/casein (n=63, with additional α-lactoglobulin and β-lactalbumin in 13 cases), followed by: wheat/gliadin and wheat/gluten (n=35 for both), *Dermatophagoides pteronyssinus*/Der p 1 (n=28), peanut/Ara h 2 (n=12), egg white/ovomucoid (n=7) and egg white/ovalbumin (n=2), apple/Mal d 1 (n=1), peach/Pru p 3 (n=2) and peach/Pru p 7 (n=1), and chicken meat/Gal d 7 (n=1).

### 3.2 Baseline characteristics

Demographic, clinical and biological data are summarized in **Table 1**. The male:female ratio was 2.02 (73:36). The median age (interquartile range (IQR)) of the study population was 8.2 years (4.2, 12.1). Ages ranged from 0.7 to 21.1 years. Forty-eight participants had BAT performed without being under allergen immunotherapy or mAb, 10 participants had BAT performed under mAb immunotherapy alone, 36 participants during allergen immunotherapy alone, and 15 participants under both allergen immunotherapy and mAb. Of note, 23/25 participants under mAb were treated by Omalizumab, and 2/25 by Dupilumab.

**Table 1:** Baseline characteristics of the study population.

Number of participants	109
Number of samples	150
Number of BAT extract/component pairs	213
Allergens (cow milk | wheat | HDM | peanut | egg white | peach | apple | chicken)	63 | 35 | 28 | 12 | 7 | 2 | 1 | 1
Demographical parameters	
Gender (M | F)^1^	73 | 36
Age, yr^1^	8.2 (4.2, 12.1)
Clinical parameters	
Under mAb^1^	25/109
Duration of mAb treatment, mo^1^	21 (12.5, 51)
Under immunotherapy^1^	51/109
Duration of immunotherapy, mo^1^	19 (8, 48)
Under mAb and immunotherapy^1^	15/109
Serological parameters	
Total IgE, kUI/L^2^	504 (156, 1191)
sIgE against extract > 0.1 kU_A_/L^2^	150/150
sIgE levels when positive (kU_A_/L) L^2^	15.7 (3.67, 95.5)
sIgG4 levels against extract, mg/L^2^	4.03 (0.36, 11.6)
sIgE against component > 0.1 kU_A_/L^3^	226/248
sIgE levels if positive (kU_A_/L) 3	7.34 (0.81, 57.1)
sIgG4 levels against component, mg/L^3^	1.1 (0.32, 4.18)

Quantitative variable are reported with median [IQR]. ^1^Among participants. ^2^Among samples. ^3^Among extract/molecular component allergen pairs. Of note, 23/25 participants under mAb were treated by Omalizumab, and 2/25 by Dupilumab.

Median (IQR) total IgE levels were 504 (156, 1191) kU/L, ranging from 3 to 12,266 kIU/L. All participants were sensitized to the tested source extracts, with median (IQR) sIgE levels of 15.7 (3.67, 95.5) kU_A_/L. All participants were also sensitized to at least one molecular allergen of the relevant source, with median (IQR) sIgE levels of 7.34 (0.81, 57.1) kU_A_/L.

Regarding BAT controls, the median (IQR) [range] of CD63^+^ basophils in negative control were 0.8% (0.4, 2.0) [0, 10.7], while median (IQR) [range] of CD63^+^ basophils was 83.5% (66.6, 89.7) [18.1, 96.7] in anti-FcϵRI positive control, and 31.1% (18.1, 49.4) [2.9, 80.3] in fMLP positive control.

### 3.3 Concordance between extract-based and allergen-based BAT

Next, we analyzed the concordance between BAT performed with extract and molecular components among 201 pairs with detectable sIgE to both extract and molecular allergen (**Table 2**). From all paired results showing sIgE sensitizations, 82% (165/201) pairs showed concordant BAT results with both extract and molecular component: 31% (63/201) were negative with both source and molecular component, and 51% (102/201) were positive with both. Cohen’s kappa coefficient was 0.62, which corresponds to moderate agreement between the two methods.

**Table 2:** Concordance between extract- and component-based BAT in component-sensitized samples from the study population.

Extract/Component results	all (n)	–/– (n)	+/+ (n)	+/– (n)	–/+ (n)	κ	p-val.	OR (95CI)
Allergen pairs								
All	201	63	102	25	11	0.63	.03	2.3 (1.1, 5.1)
Cow milk extract	83	30	32	18	3	0.51	.002	6.0 (1.8, >10)
/casein	63	27	22	13	1	0.57	.003	13 (2.0, >10)
/α-lactalbumin	7	0	5	1	1	-0.17	>.99	1 (0.01, >10)
/β-lactoglobulin	13	3	5	4	1	0.25	.37	4 (0.39, >10)
Others than cow milk	118	33	70	7	8	0.72	>.99	0.9 (0.3, 2.8)
Wheat extract (1)	70	22	42	2	4	0.81	.68	0.5 (0.04, 3.5)
/Gliadine	35	11	21	1	2	0.81	>.99	0.5 (<0.01, 9.6)
/Gluten	35	11	21	1	2	0.81	>.99	0.5 (<0.01, 9.6)
HDM	24	7	16	0	1	0.90	>.99	nc
Peanut	11	1	7	3	0	0.29	.25	nc
Egg white	9	1	5	0	3	0.27	.25	nc
/Gal d 1	7	1	4	0	2	0.36	.48	nc
/Gal d 2	2	0	1	0	1	0	>.99	nc
Peach	2	2	0	0	0	nc	nc	nc
Chicken	1	1	0	0	0	nc	nc	nc

Only pairs with positive sensitization for extract and molecular component were considered. Cohen’s kappa coefficients evaluate concordance between BAT results using component or extract as reagents. McNemar’s test evaluate unbalanced distribution of discordant results in favor of one of the two methods. Odds ratios correspond to the number of extract^+^/component^–^ over extract^–^/component^+^ results.

nc, not computed; OR, odds ratio; p-val., McNemar’s p-value; 95CI, 95% confidence interval.

Next, we investigated for any unbalance in the disagreement between the two methods. Among the 18% (36/201) discordant pairs, 13% (25/201) were extract^+^/component^–^ (69% of all discordant results), and 5% (11/201) were extract^–^/component^+^ (31% of all discordant results). This unbalance between methods in cases of disagreement was statistically significant (p=0.03), favoring positivity of extract-based BAT over component-based BAT, with the following Odds Ratio (95CI): 2.3 (1.1, 5.1). Overall, 18% (25/138) of patients with a positive BAT would have been considered negative if tested only with a molecular component against which they were nonetheless sensitized. Conversely, 8% (11/138) of BAT-positive patients would have been considered negative if tested only with the molecular component.

### 3.4 Extract-based and component-based BAT discrepancies in relation with allergic/tolerant status

Next, we sought to investigate if discordant BAT results were false-positive or false-negative results according to clinical status. Among 12 paired results from sensitized non-allergic subjects, the only discordance was an extract^-^/component^+^ discrepancy, hence allegedly a component false positive. This patient tolerated cow milk with negative BAT to cow milk (2.3%, 3.3%, and 2.9% CD63+ basophils at 1, 10, and 100 ng/mL, respectively), but presented severe anaphylaxis to goat and sheep milk and positive BAT to cow casein (19.2%, 25.0%, 23.1% CD63+ basophils at 1, 10, and 100 ng/mL, respectively).

Among 75 paired results from sensitized allergic subjects that were not under allergen immunotherapy or under mAb biotherapy, the eight discordant pairs were extract^+^/component^–^ discrepancies, hence allegedly component false negative results for milk casein (3/8), α-lactalbumin (1/8), and β-lactoglobulin (3/8), and for peanut Ara h 2 (1/8). No case of extract^-^/component^+^ discordance was observed among sensitized allergic subjects not treated by immunotherapy. These observations strongly argues in favor of the higher capacity of extract-based BAT to correlate with clinical status, as compared with component-based BAT.

### 3.5 Extract- and component-activated basophil activation rates

We hypothesized that discrepancies between extract-based and component-based BAT results could result from lower proportions of activated basophils after stimulation by similar amounts of source protein extract or molecular allergens.

**Figure 1:**
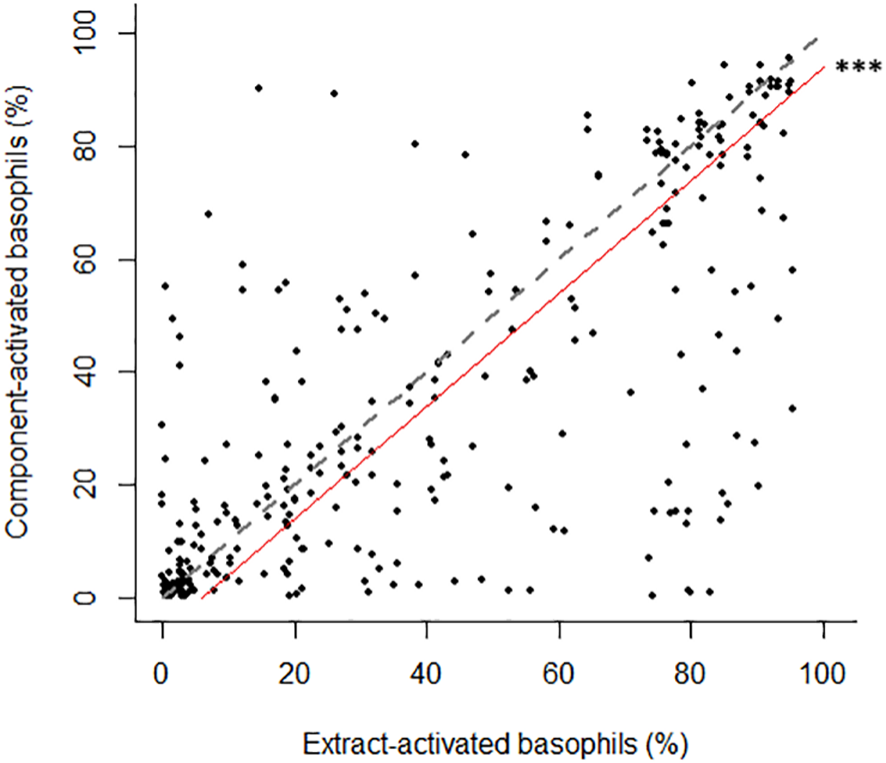
Correlation between basophil activation rates obtained with similar concentrations of molecular allergen and protein extracts from the corresponding source, from sensitized concordant BAT-positive participants from the study population. Dashed line: y=x theoretical unbiased correlation. Plain red line: correlation with slope = 1 and mean difference between dually positive pairs as the intercept. *** p-value < 0.001.

Among results from concordant positive BAT pairs from sensitized participants, extract- and component-stimulated basophil activation rates were positively correlated (Kendall’s tau-b: 0.55; p < 0.0001; **Figure 1**). However, we found significantly lower basophil activation rates with components as compared to extracts (mean difference: -6.0% with component Vs. extract; p = 0.0001).

Next, we explored if this difference between component- and extract- basophil activation rates depended of the absolute basophil activation rate. Indeed, this difference varied greatly with the basophil activation rate obtained with extract. For high basophil activation rates with extract (>80%, 60 – 80%, 45 – 60%, and 30 – 45%), component-based BAT yielded significantly lower rates (-16%, -19%, -15%, and -10%, respectively; p=2.10^-6^, 4.10^-5^,.02, and.005, respectively). For moderate (15 – 30%) basophil activation rate obtained with extract, component-based basophil activation rate were not significantly different (+2%; p=.38). For low (0 – 15%) basophil activation rate obtained with extract, component-based BAT yielded significantly higher rates (+6%; p=9.10^-5^).

We conclude that in comparison with similar amounts of source extracts, molecular components generally present lower potential to activate basophils. Nevertheless, discrepant results between the two strategies are unlikely explained solely by borderline results obtained with extract that would become negative when using components.

### 3.6 BAT discordances and considered allergenic source

Because the discrepancies observed between extract- and component-based BAT were unlikely to be entirely explained by the lower propensity of molecular components to trigger basophil activation, we stratified our study population by allergen source and performed a concordance and disagreement study.

Concordance and disagreement per extract/molecular allergen pairs are reported in **Table 2**. Results revealed low agreement between extract- and component-based BAT with cow milk (kappa = 0.51), especially for casein (kappa = 0.57). However, overall good agreement was reached for other allergens (kappa = 0.72). Similarly, the study of the discrepancies confirmed that extract-based BAT was more sensitive than component-based BAT for cow milk (p = 0.002), and for casein (p = 0.003). Regarding other allergens, discordant results were as likely to be extract+/component– or extract–/component+ (p>0.99).

Next, we investigated the difference between extract- and component-stimulated basophil activation rates, according to allergen source, using a linear regression model (**Table 3**). This model confirmed lower basophil activation rates with milk-component (p < 0.0001), but also with peanut and Ara h 2 (p <0.0001). In contrast, egg- (p < 0.0001) and wheat-components (p = 0.01) yielded higher basophil activation rates than their extract counterparts. Other allergens did not show significant differences regarding basophil activation rates with extract or molecular components.

**Table 3:** Association between allergen source and the differences in percentage of basophil activation using molecular component against source extract.

Allergen	β-coefficient (± sd)	p-val.
**Cow milk**	**– 4.90 (**± **1.12)**	**< 0.0001**
**Wheat**	**+ 4.31 (**± **1.68)**	**0.011**
HDM	**–** 2.95 (± 2.28)	ns
**Peanut**	**– 26.01 (**± **3.24)**	**< 0.0001**
**Hen egg**	**+ 15.79 (**± **3.68)**	**< 0.0001**
Peach	+ 3.36 (± 10.58)	ns
Apple	+ 3.00 (± 18.26)	ns
Chicken meat	+ 5.10 (± 8.23)	ns

The table display unadjusted β-coefficients corresponding to the difference in percentage of activated basophils between molecular components and source extract. These coefficients were determined by conducting multicategorical univariable linear regression model using BAT results from all allergen dilutions coming from concordant extract^+^/component^+^ BAT positive results from the study population. Bold lines correspond to allergen that associated with significantly lower or higher differences in percentage of activated basophils with molecular component compared to source extract.

BAT, basophil activation test; HDM, house dust mite; ns, not significant; p-val., p-value; sd, standard deviation.

We conclude that the respective performances of extract-based and component-based BAT likely depend of the considered allergen.

### 3.7 Other factors associated with extract/component BAT discordances

Finally, we examined if any demographic (age, sex), clinical (definite allergy, presence and duration of mAb and/or allergen immunotherapy) or biological variable (total IgE, specific IgE, specific IgG4, BAT negative and positive controls) was associated with higher risk of discordant BAT result, independently of the considered allergen source. In order to do so, we conducted multivariable logistic regression analyses of discordant pairs against concordant positive pairs. Results are summarized in **Table 4**.

**Table 4:** Adjusted associations of discordant BAT results with demographic, clinical, and biological variables.

Variables	Extract +/Component –		Extract –/Component +	
	aOR (95CI)	p-val.	aOR (95CI)	p-val.
Demographic variables				
Male gender (male)	–	ns	–	ns
Age (per +10 yr)	–	ns	1.13 (1.01, 1.28)	0.04
Clinical variables				
Definite allergy (yes)	–	ns	–	ns
mAb immunotherapy (yes)	–	ns	1.23 (1.07, 1.41)	0.004
Duration of mAb immunotherapy (+ 1 yr)^1^	–	ns	–	ns
Allergen immunotherapy (yes)	–	ns	1.19 (1.06, 1.34)	0.004
Duration of allergen immunotherapy (+ 1 yr)^1^	–	ns	0.94 (0.90, 0.99)	0.03
Serological variables				
total IgE (per + 1000 kU/L)	1.08 (1.01, 1.15)	0.03	1.08 (1.04, 1.13)	0.0001
extract sIgE (per + 10 kU_A_/L)	–	ns	–	ns
component sIgE (per + 10 kU_A_/L)	–	ns	–	ns
extract sIgG4 (per + 1 mg/L)	–	ns	–	ns
component sIgG4 (per + 1 mg/L)	–	ns	–	ns
BAT variables				
negative control (per + 10%)	–	ns	–	ns
FcϵRI-stimulated positive control (per + 10%)	–	ns	1.05 (1.00, 1.05)	0.048
fMLP-stimulated positive control (per + 10%)	–	ns	1.03 (1.01, 1.06)	0.017

The table display adjusted odds ratios conveying higher risk of presenting the corresponding discordant result against presenting concordant extract^+^/component^+^ BAT positive result in the study population, obtained by multivariable logistic regression models. All models were adjusted for allergen source (cow milk, wheat, HDM, hen egg, peanut, apple, peach, chicken meat). Of note, similar results for total IgE and mAb therapy were found in additional models adjusted against each other. The risk associated with duration of mAb immunotherapy and duration of allergen immunotherapy was calculated among patients under mAb or allergen-specific immunotherapy. Only statistically significant adjusted odds ratios are displayed.

^1^Computed among patients under mAb or allergen-specific immunotherapy, respectively

aOR, adjusted odds ratio; BAT, basophil activation test; ns, not significant; p-val., p-value; sIgE, specific IgE; sIgG4, specific IgG4; yr, years; 95CI, 95% confidence interval.

Independently of the allergen source, a higher risk of discordant results (either extract+/component– or extract–/component+) was associated with higher total IgE levels (p = 0.03, and 0.0001, respectively). In addition, higher risk of presenting extract–/component+ BAT results was associated with higher age (p = 0.04), higher BAT positive control results (p = 0.048 and 0.017 for FcϵRI and fMLP, respectively), and with mAb-associated (p = 0.004) or allergen-specific immunotherapy (p = 0.004). Interestingly however, among patients under allergen immunotherapy, the duration of immunotherapy was associated with lower risk of discordant BAT results (p = 0.03), meaning higher discordant rate during the early phase allergen-specific immunotherapy. Of note, the significant association of discordant extract–/component+ BAT results was observed for Omalizumab (p=0.001, aOR 1.28 (CI95:1.11, 1.49), n=46), but not with samples under Dupilumab (p>0.99, aOR 1.00 (CI95:0.74, 1.36), n=3). In addition, similar results were found for mAb therapy when adjusting for total IgE (and reciprocally) in additional models.

We conclude that, in addition to the considered allergen and molecular component, several factors likely contribute to discordant BAT results.

## 4 Discussion

### 4.1 Main results

In this monocentric retrospective systematic study, paired extract- and component-based BAT results for various food allergens in children showed only a moderate agreement (κ = 0.62), and 18% (36/201) discordances. Among 36 cases of discrepant BAT results, almost 7 out of 10 were extract^+^/component^–^, rather than extract^–^/component^+^ (p=0.03). Additionally, among sensitized tolerant subjects, or genuinely allergic patients untreated by immunotherapy, all 9/9 BAT discordant cases showed that extract-based BAT, and not component-based, was in line with patient’s clinical status. Even among extract^+^/component^+^ concordant BAT cases, component-stimulated basophils showed 6% less activation rates than their extract-stimulated counterpart. However, our data suggest that these findings might be modulated by the considered allergen, and mainly due to the milk/casein pairs in this cohort. Independently of the allergen, on other factors were associated with extract/component discrepancies, such as age, total IgE, BAT positive control values, and treatment by mAb biotherapy or allergen-specific immunotherapy.

### 4.2 Strengths and limitations

One of the main limitation of this study is its monocentric retrospective design. In order to minimize additional selection biases, we systematically reviewed all BAT results prescribed and performed in our pediatric tertiary allergy center. Potential selection biases would only be limited to referral bias to the center, and hypothetical prescription bias of paired extract- and component-based BAT, instead of extract-based BAT only, for instance toward more unusual presentations or cases with difficult diagnosis. Regarding that matter, it is not excluded that the frequency of discordant results could have been overestimated. In the other hand, given the study design, BAT prescriptions from this cohort are expected to closely reflect real-life cases in which the need for both extract- and component-BAT results were prescribed by experienced physicians.

One of the strengths of the study lies in the exhaustive analysis of paired extract- and component-based BAT results among the same individuals. This study constitutes, to the best of our knowledge, the largest series of this type to date, encompassing multiple food and pneumallergens sources and components, from various clinical situations: at baseline, during mAb, and/or during allergen immunotherapy. In addition, we took care to limit our analyses to extract- and component-sensitized pairs, in order to limit discordances related to the absence of sIgE. This point is overlooked in many BAT studies, where non-sensitized individuals often constitute all, or part, of the reference groups.

Other limitations of the study lie in the nature of the tested allergens: mainly foods, with unbalanced group sizes. In order to limit heterogeneity in BAT interpretation for different sources, we restricted to manufacturer’s recommendations, and used fixed BAT parameters (same activation marker (CD63), similar allergen concentrations for component and extract, fixed decision thresholds), rather than operate with *ad hoc* thresholds per allergen (10).

### 4.3 Discussion

There is only scarce literature studying the direct comparison of extract- and component-BAT performances. In most studies, extract- and component-BAT performances were compared with optimal *ad hoc* cut-offs, sometimes with different allergen concentrations, which prevent direct comparison of extract and molecular component potency to activate basophils (11–15). In line with our results, however, optimal thresholds were lower with component- than with extract-based BAT, for milk (11, 12), egg (11), wheat (13) or peach (14). Similarly, the threshold recommendations from the BAT kit manufacturer are 15% for extracts, vs 10% for wheat and peanut molecular allergens, which is consistent with a lower capacity of single molecular allergens to activate sensitized basophils.

From the mechanistic standpoint, lower sensitivity of basophils to a single molecular allergen as compared to similar amounts of protein extract could be explained by several reasons. First, some component-specific IgE might not be able to activate basophils, corresponding to “clinically irrelevant IgE”. Second, recombinant components might not be as efficient as purified native molecular allergens to activate basophils, as illustrated by the case of native and recombinant ω-5-gliadin in the study by Tokuda et al. (13) Third, IgE-mediated FcεRI bridging might be facilitated by a mixture of allergens and protein complexes, as suggested by the work of Hemmings et al. where peanut IgE diversity potentiated effector cell activation (16). Fourth, non-allergenic proteins within source extract could also potentiate basophil activation through IgE-independent pathways, such as Lectin receptors- (17), MRGPRX2- (18), or PRR-dependent pathways (19).

The practical implication of lower basophil activation by single molecular allergen have however to be discussed. Firstly, lower (or higher) activation rate does not necessarily entail lower (or better) diagnostic performances of component-based BAT, especially if performed with adapted allergen concentrations and optimal cut-offs. To our knowledge, no study clearly demonstrated significant differences in diagnostic sensitivity or specificity between extract and molecular allergens (11–15). However, the use of *ad hoc* optimized parameters in the absence of validation dataset in these studies impedes precise estimation and comparison of close diagnostic performances. Our results confronting BAT discordances with sensitized-tolerant or untreated-allergic status suggest that extract-based BAT may display better diagnostic performances, only with similar allergen concentration and decision points (except for wheat proteins and Ara h 2, where 10% CD63^+^ basophil activation was used as positivity threshold, as per manufacturer’s recommendations).

Secondly, the lower sensitivity of basophils to molecular allergens may not apply to every allergen sources, nor to every molecular component from a source. For instance, unlike cow milk and casein, egg white- and ovomucoid- did not show significant dissociation in our cohort, in line with the findings by Sato et al. in milk and egg allergy (11).

Third, in specific situations, component-based BAT could provide more accurate information as compared to extract-BAT. This might be the case for the diagnosis of systemic reactions, such as LTP-related reactions to fruit and vegetables (14, 15, 20), where extract-based BAT would also convey PR10-related positivity associated with oral allergy syndrome; for the diagnosis of specific reactions to cooked or processed foods, for instance casein-BAT as a surrogate for baked milk BAT; for the diagnosis of reactions to molecular allergen that are usually present in low abundance or unstable in protein extract, such as PR10 (21), as illustrated for soy allergy (22).

### 4.4 Conclusion

In conclusion, this monocentric retrospective systematic study on 213 extract/component pairs revealed a moderate agreement and a high rate of discordances between the two reagents. Importantly, in discrepant cases, BAT results obtained with extract conveyed more accurately patient’s clinical status. Single molecular allergens induced lower basophil activation rates as compared with similar amounts of source protein extract. Because this effect was mainly associated with the milk/casein pairs in the present cohort, further studies are needed to validate these findings in different allergens, and to account for the effect of other sociodemographic, clinical, and biological variables. In practice, if both cannot be performed, we suggest that BAT should be preferentially performed with protein extract alone, rather than with molecular allergen alone.

## Data Availability

The datasets presented in this article are not readily available because of local institution policies. Requests to access the datasets should be directed to yannick.chantran@aphp.fr.
